# Supplementary material for: Antioxidant Activity of Aqueous Extracts Obtained from By-Products of Grape, Olive, Tomato, Lemon, Red Pepper and Pomegranate
Source: Foods. 2024 Jun 7;13(12):1802. doi: 10.3390/foods13121802 (PMC11202578; doi:10.3390/foods13121802)
Supplement: Supplementary file 1 [file foods-13-01802-s001.zip › foods-3014170-supplementary.pdf]

Figure S1.- Chemical structure of the identified compunds (source: https://pubchem.ncbi.nlm.nih.gov/)

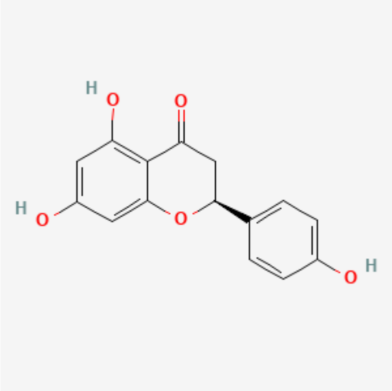

Figure a.- Naringenine

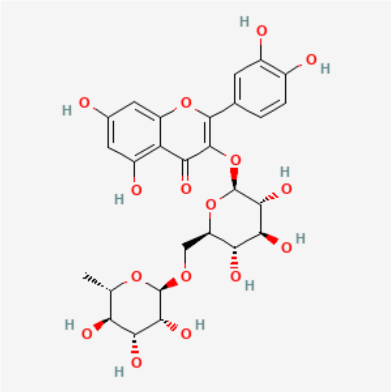

Figure b.- Rutine

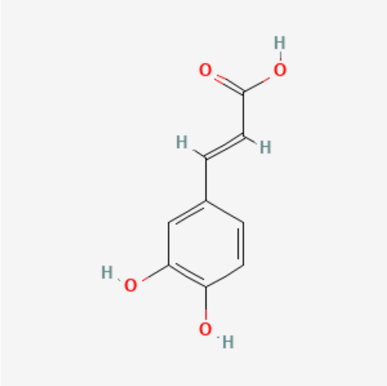

Figure c.- Caffeic acid

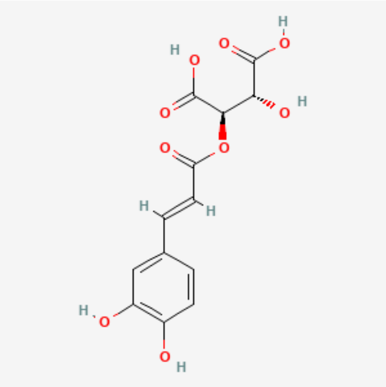

Figure d.- Caftaric acid

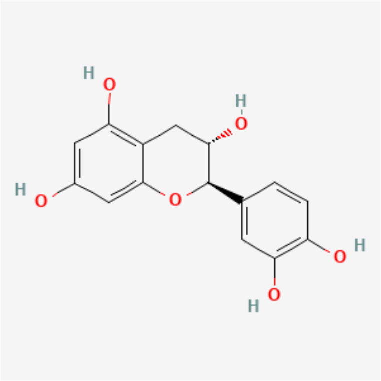

Figure e- Catechin

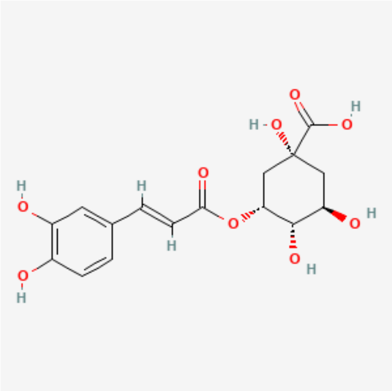

Figure f.- Neochlorogenic acid

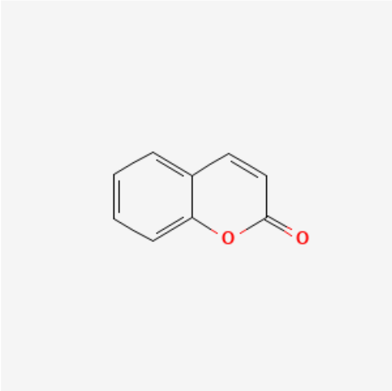

Figure g.- Coumaric acid

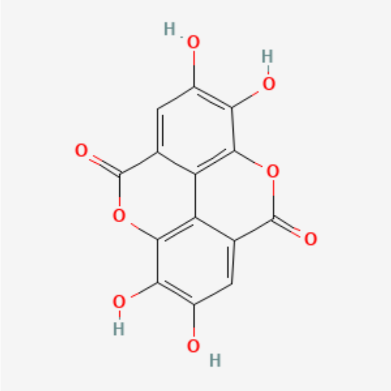

Figure h.- Ellagic acid

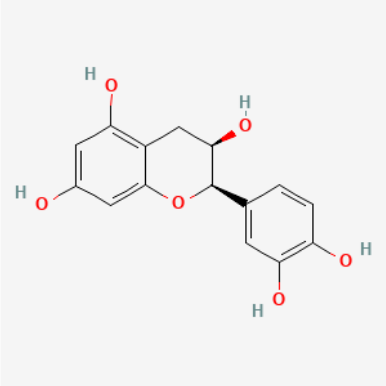

Figure i.-Epicatechin

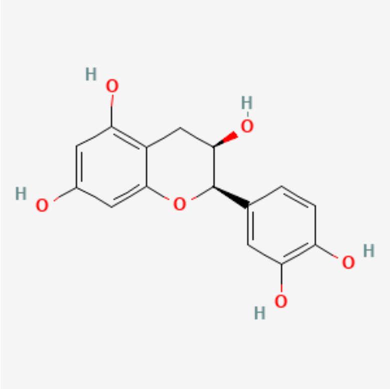

Figure j.-4-Hydroxybenzoic acid

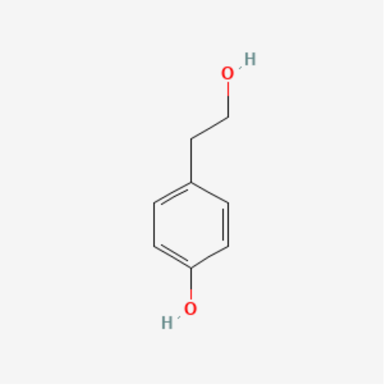

Figure k.-2,4-Hydroxyphenil Ethanol

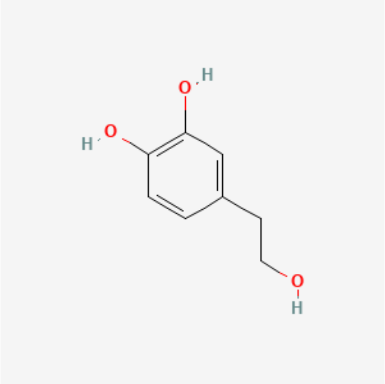

Figure l.-Hydroxytyrosol

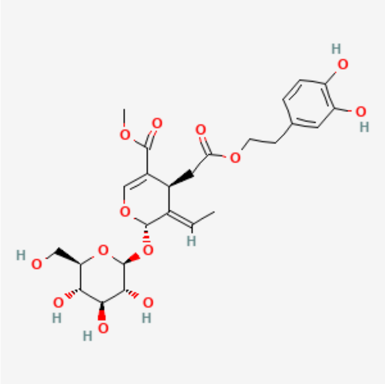

Figure m.-Oleuropein

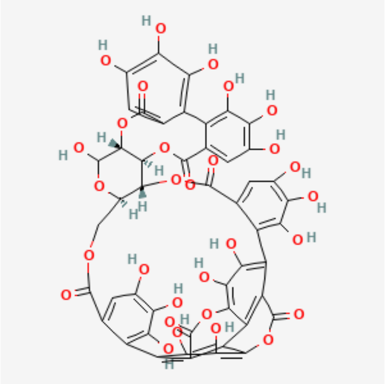

Figure n.-Punicalagin

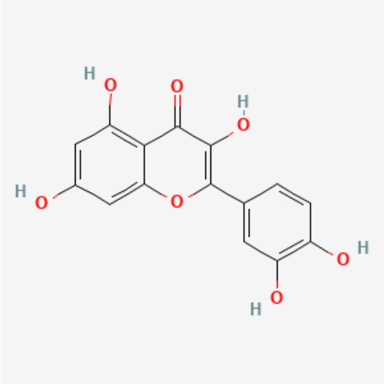

Figure o.- Quercetin

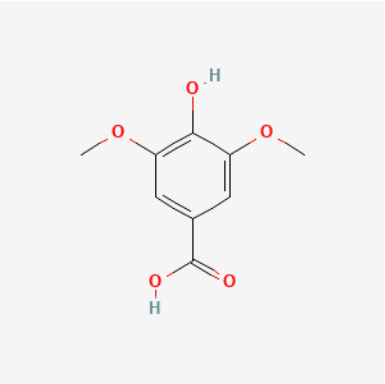

Figure p.- Syringic acid

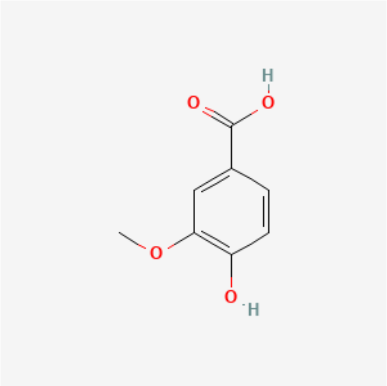

Figure q.- Vanillic acid

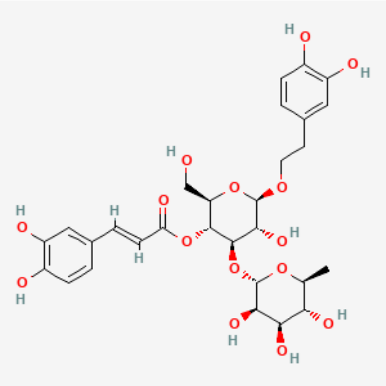

Figure r.- Verbascoside
